# Development of Pectin and Poly(vinyl alcohol)-Based Active Packaging Enriched with Itaconic Acid and Apple Pomace-Derived Antioxidants

**DOI:** 10.3390/antiox11091729

**Published:** 2022-08-31

**Authors:** Bernadette-Emőke Teleky, Laura Mitrea, Diana Plamada, Silvia Amalia Nemes, Lavinia-Florina Călinoiu, Mihaela Stefana Pascuta, Rodica-Anita Varvara, Katalin Szabo, Patricia Vajda, Cristian Szekely, Gheorghe-Adrian Martău, Simon Elemer, Floricuța Ranga, Dan-Cristian Vodnar

**Affiliations:** 1 Life Science Institute, University of Agricultural Sciences and Veterinary Medicine Cluj-Napoca, 400372 Cluj-Napoca, Romania; 2 Faculty of Food Science and Technology, University of Agricultural Sciences and Veterinary Medicine, Cluj-Napoca, Calea Mănăștur 3-5, 400372 Cluj-Napoca, Romania

**Keywords:** biopolymer, organic and phenolic extract, lyophilization, itaconic acid, antioxidant activity, apple pomace, viscosity, water vapor permeability, water solubility

## Abstract

The production of active and biodegradable packaging materials is an emerging and efficient alternative to plastic packaging materials. By combining poly(vinyl alcohol) (PVA), pectin, and itaconic acid (IA), biodegradable and water-soluble packaging materials can be obtained that can also increase the shelf-life and quality of foodstuff. In the present study, the generated film-forming solutions were enriched with organic or phenolic extracts from apple by-products (apple pomace). These extracts possess an efficient antioxidant activity of 9.70 ± 0.08, and 78.61 ± 0.24 μM Trolox/100 g fresh weight, respectively. Furthermore, the lyophilization of these by-products increased the extract’s organic and phenolic content and the antioxidant activity to 67.45 ± 0.28 and 166.69 ± 0.47 μM Trolox/100 g fresh weight, respectively. These extracts influence the physical-chemical properties of the biofilm solutions by facilitating the polymerization process and thus positively influencing their viscosity. The resulting biofilms presented low water vapor permeability and reduced solubility in water. Adding IA and organic/phenolic compounds facilitates the resistance against intrinsic and extrinsic factors; therefore, they might be applicable in the food industry.

## 1. Introduction

Plastic and plastic-based packaging rise serious pollution concerns worldwide regardless of their consistent contribution to the global economy [1]. Nowadays, worldwide plastic production is at 320 million (M) tons per year, with a growing demand, indicating its broad-spectrum applicability in various areas [2]. Food packaging accounts for half of all polymers produced from fossil fuels in the packaging sector [3]. These fossil fuel-derived polymers are persistent in the environment, taking hundreds of years to decompose, thus contributing to freshwater and ocean pollution [4,5]. Based on the “Plastics 2030 Voluntary Commitment” objectives, it is crucial to reach a 60% reuse, recycling, and reduction of plastics until the year 2030, and 100% as far as 2040 [6]. Consequently, there is an increasing demand for environmentally friendly, sustainable packaging materials with appropriate quality characteristics [7,8,9]. According to the European Bioplastics market, the bio-based and biodegradable polymer production capacity is expected to increase from roughly 2.11 M tons in 2018 to 7.59 M tons in 2026 (https://www.european-bioplastics.org/market/, accessed on 25 August 2022). With regards to food packaging, these bio-based and biodegradable polymers have an active role in conserving and shielding the food product from the external environmental stressors. Given the numerous advantages of these polymers, the production of active packaging with antioxidant effects impacts positively the shelf-life of foodstuff, and improve their quality [10,11,12].

Itaconic acid (IA) is an essential chemical for producing polymers that originates from renewable resources. IA has been manufactured industrially since the mid-twentieth century using the filamentous fungus *Aspergillus terreus*. Still, several microorganisms are genetically engineered to synthesize this organic acid in significant quantities and on various substrates [7,13]. It is recognized as one of the twelve most significant biomass-derived molecules capable of being converted into a variety of useful chemicals or substances [14]. IA’s chemical structure—one unsaturated double bond and two carboxyl groups—enables the production of a wide range of innovative biopolymers, including drug carriers, antibacterial biopolymers, and, most notably, intelligent food packaging [7,13,15]. Several studies analyze the effect of IA on bacterial growth inhibition, energy metabolism, and its immunomodulatory properties, but there are still gaps regarding itaconate biology [16,17].

Poly(vinyl alcohol) (PVA) is a water-soluble synthetic polymer with high structural capacity, strong compound characteristics, good biodegradability, and ease of usage. The efficient physical properties of PVA are attributed to its hydroxyl groups, which support the creation of hydrogen bonds [18]. Due to their abundance on the surface, it is one of the most hydrophilic polymers with high moisture sensitivity. As a result, its associated blends and composite materials have become popular for packaging applications [19]. PVA has been investigated in several studies as a possible biopolymer to replace traditional plastic materials in various uses, particularly in packaging. PVA may be used for film development for its strong film-forming ability, biodegradability, crystal modulus, and increased crystallinity [20,21].

Pectin is a branched heteropolysaccharide composed of long-chain galacturonan segments toghether with other neutral sugars, including galactose, arabinose, and xylose [22]. Pectin has several applications, and is primarily used in the food industry as emulsifier, stabilizer, gelling agent, thickener, and fat or sugar replacer in low-calorie meals [23]. Pectin and pectin-derived oligosaccharides can also be significant elements in functional food development and food packaging [24,25]. Pectin-based packaging provides various advantages in improving the shelf-life of food products by slowing lipid oxidation, inhibiting microbial activity, and regulating water movement [26]. Active packaging with antioxidant properties is an efficient alternative to conventional packaging, which can decrease the food’s oxidative damage, one of the leading causes of food spoilage.

Apple (*Malus domestica* Borkh) is one of the world’s most popular fruits due to its great flavor and nutritional content, ranking fourth in fresh fruit consumption globally [27]. According to data from the United Nations Food and Agriculture Organization (FAO), the world’s approximate supply of apples in 2017 was 81M tons (FAOSTAT, 2017). One third of the cultivated apples (79M bushels) are processed into juices, ciders, alcoholic drinks, sauces, canned apples, dried apples, and frozen apple slices. As apples are processed, 25% of the fruit mass (e.g., skin, stem, seeds, and pulp) is abandoned as waste, referred to as apple pomace (AP) [28]. Given the high volume of the generated by-products during juice production and processing, commercial pomace applications could significantly impact the economy. Considerable progress has been made in converting food processing and agriculture waste into economically viable goods and value-added products such as bio-fuels, nutrients, and multifunctional additives [29,30]. Consequently, numerous applications of AP have been studied in the recent years to increase its recovery rate [31]. The investigation of apple by-products is a vast research area given the wide variety of organic and phenolic compounds, main bioactive compounds, the most appropriate extraction methods, purification techniques, biological applications, and analysis techniques in food packaging [32].

The current study intended to analyze the recent progress in IA-based material production and the subsequent synthesis of biodegradable polymers, together with their applicability in the food packaging industry. In addition, the need to substitute polymers derived from the petrochemical industry and recover and reuse waste materials is a topical subject. This study aimed to develop IA-based active films using a blend of PVA, pectin, and enriched with AP extracts (organic and phenolic) for food packaging applications while determining its morphologic and mechanical properties. Moreover, the extract’s antioxidant and antimicrobial activity and their effect on film-forming solutions, and rheological characteristics have also been determined.

## 2. Materials and Methods

### 2.1. Materials

Throughout the experiments, the used reagents were of analytical grade, such as ethanol, acetic acid, DPPH (2,2-diphenyl-1-picrylhydrazyl), Trolox (6-hydroxy-2,5,7,8-tetramethylchroman-2-carboxylic acid), and pectin from apple were acquired from Sigma-Aldrich (Steinheim, Germany). For biofilm preparation, the used PVA was of high molecular weight (98–99%) hydrolyzed; it was obtained from ThermoFisher (Kandel, Germany). For the antimicrobial and antioxidant assays, the used chemicals, Mueller–Hinton broth and agar, peptone, tryptic soy broth, and resazurin were acquired from BioMerieux (Marcy l’Etoile, France).

Chemical reagents and materials for HPLC. NaH_2_PO_4_ was provided by Merck (Darmstadt, Germany), and water was purified with a Direct-Q UV system by Millipore (St. Louis, MO, USA). The pure standard of malic, ascorbic, citric, succinic, and fumaric acid (purity 99% HPLC) was purchased from Merck, Germany. Acetonitrile, HPLC-gradient, was provided by Merck (Darmstadt, Germany) and water was purified with a Direct-Q UV system by Millipore (St. Louis, MO, USA). The pure standard of cyanidin, catechin, rutin, gallic and chlorogenic acid (99% HPLC) were purchased from Sigma (St. Louis, MO, USA). Apple pectin was procured from Sigma^®^ (Darmstadt, Germany). Calcium chloride (CaCl_2_) and glycerol were bought from VWR Chemicals. CaCl_2_ anhydrous was procured from Cristal R Chim SRL, Romania.

### 2.2. Preparation and Characterization of Apple Pomace Extracts

The apple variety selected for this study was Red Delicious, bought from a local market (Cluj-Napoca, Romania). AP was prepared in the University of Agricultural Science and Veterinary Medicine Cluj-Napoca biotechnological laboratory. Phenolic compounds can act as a substrate in enzymatic browning, primarily caused by polyphenol oxidase (PPO). As a result, AP is a very vulnerable substrate to oxidation processes [33]. For the inactivation of PPO, the apples were immersed in a water solution with 1% ascorbic acid and 1% citric acid at 95 °C for 4 min. After PPO’s inactivation, the apple juice and AP were separated using an apple juice machine (Gorenje, JC800G, PRC). For a lower water content, gauze was used.

#### 2.2.1. Freeze-Drying Process

The apparatus used for the freeze-drying process was a Telstar LyoQuest -55 Plus with a condenser at −55 °C (Terrassa, Azbil Group, Spain). Apple by-products were added in a ratio of 1:3 into a 1000 mL capacity flask and were immediately frozen at target temperatures (−80 °C) for 24 h. After freezing, the samples were placed in the lyophilization apparatus and kept for nearly 72 h. The parameters for the drying process were set to 0.001 mBar vacuum and −55 °C temperature. The samples were dried until they reached a constant weight (±0.005 g). The water content evaporated by the freeze-drying process is expressed as a percentage (%), considering the mass before and after drying [34].

#### 2.2.2. Organic Acid Extraction and HPLC

**Sample preparation.** A 1 g sample with 5 mL distilled water was vortexed for 1 min, followed by 15 min ultrasonic treatment at room temperature, then centrifuged at 10,000 rpm for 10 min. The supernatant, containing extracted organic acids, was filtered through a nylon filter (pore size 0.45 μm), and 20 μL was injected into the high-performance liquid chromatography (HPLC) system.

**Chromatographic condition.** The HPLC analysis was performed utilizing an HP-1200 liquid chromatograph equipped with a quaternary pump, manual injector and VWD detector (Agilent-Technologies, USA, Santa Clara, CA, USA). The column was an Acclaim OA (5 μm; 4 × 150 mm i.d.) from Thermo Fisher Scientific, Waltham, MA, USA. The mobile phase was NaH2PO4 50mM solution at pH = 2.8, elution was done for 10 min at room temperature with a flow rate of 0.5 mL/min. Chromatograms were recorded at wavelength λ = 210 nm, and data acquisition was made with the Agilent ChemStation software.

**Quantitative determination.** The organic acid content was determined using a five-point calibration curve for each organic acid. The chromatograms of standards, frozen AP, and lyophilized AP are presented in Supplementary Figure S1.

#### 2.2.3. Extraction and HPLC-DAD-MS-ESI ^+^ Analysis of Phenolic Compounds

**Sample preparation.** A 1 g sample with 5 mL methanol was vortexed for 1 min, followed by 15 min ultrasonic treatment at room temperature. The supernatant, containing extracted polyphenols, was filtered through a nylon filter (pore size 0.45 μm) and 20 μL was injected into the HPLC system [35].

The 1 g frozen sample, respectively, 0.25 g of dry lyophilised samples, were mixed with 5 mL ethanol 70%, vortexed for 1 min followed by 15 min ultrasonic treatment at room temperature, then centrifuged at 10,000 rpm for 10 min at 40 °C. The supernatant was collected and kept in a dark place. Over the pellet, the solvent was re-added, and the sonication-centrifugation procedures were repeated 3 times.

The extract collected after each centrifugation was concentrated by evaporation under low pressure (150 mbar), at low temperature at 35 °C, and 180 rpm with a rotary evaporator (Heidolph rotary evaporator, Schwabach, Germany) [36] to a volume of 5 mL, then filtered through a nylon filter (pore size 0.45 μm), and 20 μL was injected into the HPLC system.

**Chromatographic condition.** Analysis was carried out using an HP-1200 liquid chromatograph equipped with a quaternary pump, autosampler, DAD detector, and MS-6110 single quadrupole API-electrospray detector (Agilent-Technologies, USA). The positive ionization mode was applied to detect the phenolic compounds; a different fragmentor was applied in the range of 50–100 V. The column was a Kinetex XB-C18 (5 μm; 4.5 × 150 mm i.d.) from Phenomenex, Torrance, CA, USA.

The mobile phase was (A) water acidified by acetic acid 0.1% and (B) acetonitrile acidified by acetic acid 0.1%. The following multistep linear gradient was applied: start with 5% B for 2 min; from 5% to 90% of B in 20 min, hold for 4 min at 90% B, then 6 min to arrive at 5% B. The total time of analysis was 30 min, flow rate 0.5 mL/min, and oven temperature 25 ± 0.5 °C

Mass spectrometric detection of positively charged ions was performed using the Scan mode. The applied experimental conditions were: gas temperature 3500 °C, nitrogen flow 7 L/min, nebulizer pressure 35 psi, capillary voltage 3000 V, fragmentor 100 V, and *m*/*z* 120–1500. Chromatograms were recorded at wavelength λ = 280, 340, and 520 nm and data acquisition was done with the Agilent ChemStation software.

Quantitative determination. The hydroxybenzoic acid and dihydrochalcone content were determined using a five-point calibration curve of gallic acid at 280 nm (R^2^ = 0.9978) in the linearity range of 10–100 μg/mL. The hydroxycinnamic acid content was determined using a five-point calibration curve of chlorogenic acid at 340 nm, (R^2^ = 0.9937) in the linearity range 10–50 μg/mL. The flavonol content was determined using a five-point calibration curve of rutin at 340 nm (R^2^ = 0.9981) in the linearity range of 10–100 μg/mL. The anthocyanin content was determined using a five-point calibration curve of cyanidin at 520 nm (R^2^ = 0.9951) in the linearity range of 10–100 μg/mL. The flavanol content was determined using a five-point calibration curve of catechin at 280 nm (R^2^ = 0.9994) in the linearity range 10–100 μg/mL.

### 2.3. Antioxidant Activity of Extracts before and after Lyophilization

The antioxidant activity of the samples was determined before and after lyophilization using the DPPH (1,1-diphenyl-2-picrylhydrazyl) free radical scavenging capacity technique developed by Brand-Williams et al. [37]. To determine the samples’ antioxidant responses, they were prepared in triplicate, 35 μL of previously aqueous extracted samples was mixed with 250 μL of methanolic DPPH solution. The reaction solution was incubated for 30 min at room temperature in the darkness before measuring absorbance at 515 nm using a multi-mode plate reader (BioTek, Winuschi, VT, USA). The findings were presented as micromol Trolox equivalents (μmol TE)/100 g sample.

### 2.4. Film Preparation

#### 2.4.1. PVA Films

The biofilm solutions were prepared by developing a base solution (PVA+Gly), the control sample. This solution was made with 3% wt. PVA and Gly (30% *w*/*w* dry mass PVA) dissolved in hot distilled water (90 °C) under constant stirring for two hours. Further, the solvent casting technique was used to prepare the polymer films, as indicated by Szabo et al. in their study [20]. To the base solution (PVA+Gly) 1% *v*/*wt*. IA was added under stirring conditions and at a temperature of 65 °C for 30 min, to obtain the formulation PVA+Gly+IA. This solution was enriched with two different AP extracts (9% phenolic extract (PE) and organic extract (OE)), resulting in two other formulations (PVA+Gly+IA+PE and PVA+Gly+Ia+OE). The extracts were incorporated into the PVA+Gly+IA solution under continuous stirring for 30 min at room temperature (21 °C). Further, all the biofilm solutions were analyzed regarding their antimicrobial activity and rheological measurements. Then, the process continued with the casting of the biofilm solutions into Petri dishes, and they were left for solidification at room temperature (21 °C) for 48 h. The solidified biofilms were peeled off and subjected to several investigations (e.g., physical measurements, water vapor permeability, etc.).

#### 2.4.2. Pectin Films

Films were prepared according to [38] and adapted to our application. IA (1% *w*/*w*) was dissolved under a gentle stirrer in pre-heated distilled water (75 ± 5 °C) for 1 h. The solution temperature was kept to 75 ± 5 °C. Then, Gly (30% *w*/*w* dry mass pectin) and CaCl_2_ (1% *w*/*w* dry mass pectin) were added. The whole mixed solution was homogenized to 9500 rpm for 5 min (Ultraturax Polytron PT 6100 D, Kinematica, PT-DA 20/2 EC-F193) while pectin (2% *w*/*w*) was gradually added. The homogenization was continued for another 5 min to 15,000 rpm using Ultraturax. When necessary, apple extract (PE and OE) was incorporated into the obtained mixed solution at 15,000 rpm for 5 min. Then, the mixed solution was kept at room temperature (25 °C) until the next day for better pectin hydration. The foam was removed with a spoon, and the mixed solution was immersed into the ultrasonic water bath (Elmasonic E 15 H, 50/60 Hz, Elma) at 55 ± 5 °C for a minimum of 30 min to remove air bubbles. Films were obtained using the casting method. A total of 15 g of a mixed solution were poured into Petri dishes (9 cm in diameter) and stored at room temperature for a minimum of 4 days before further analysis. Control films were obtained from pectin, glycerol, and CaCl_2_ following the same procedure.

### 2.5. Antimicrobial Activity of Extracts and Biofilm Solutions

#### 2.5.1. Standard Strains

The following seven standard strains were tested: *Escherichia coli* ATCC 25922, *Staphylococcus aureus* ATCC 29213, *Staphylococcus epidermidis* ATCC 12228, *Pseudomonas aeruginosa* ATCC 27853, *Enterococcus faecalis* ATCC 29212, *Streptococcus pyogenes* ATCC 12344, and *Salmonella enterica* NCTC 6017. The strains were grown in a test tube containing 10 mL sterile Mueller–Hinton broth (Oxoid Ltd., Basingstoke, Hampshire, England), Tryptone Soya broth (CM0129, Thermo Fisher Scientific, USA) and Brain Heart Infusion broth (M210, HiMedia Laboratories, Pvt. Ltd.), respectively, at 37 °C for 24 h. A loopful of grown inoculum was streaked on growth agar medium: Tryptic Soy agar (M1968, HiMedia Laboratories, Pvt. Ltd.) for *E. coli* and *P. aeruginosa*, Mueller–Hinton agar (Oxoid Ltd., Basingstoke, Hampshire, England) for *S. epidermidis*, *S. aureus*, *S. enterica*, and Brain Heart Infusion agar for *E. faecalis* and *S. pyogenes*. Plates were incubated for 24 h at 37 °C. Bacterial morphology was confirmed by optical microscopy.

#### 2.5.2. Preparation of Bacterial Strains

Several colonies of each strain cultivated on the media were transferred in 9 mL of sterile NaCl solution (8.5 g/L) and adjusted to match the turbidity of McFarland 0.5 standard (1.5 × 10^8^ CFU/mL). Then, a 1.5 × 10^5^ CFU/mL bacterial suspension was prepared to be added to each microplate.

#### 2.5.3. Determination of the Minimum Inhibitory Concentration (MIC)

The MIC was determined using the resazurin microtiter plate-based antibacterial assay [39,40,41]. One hundred µL of sterile specific growth broth medium was added to the wells of a 96-well microplate. Then, 100 µL of each extract and obtained biofilms were added in the first wells, and serial 11-fold dilutions were made in the subsequent wells of each row by transferring 100 µL from well to well. The surplus of 100 µL in the last well of the row was discarded. Then, 10 µL of inoculum (1.5 × 10^5^ CFU/mL) was added to all the wells. The positive control was gentamicin (0.4 mg/mL in saline solution). The biofilm solutions without the extracts were added as a negative control.

The microplates were incubated for 20–22 h at 37 °C, and 20 µL of 0.2 mg/mL resazurin aqueous solution was added to all the wells. The microplates were subjected to a subsequent 2 h incubation at 37 °C. After this period, resazurin (a blue non-fluorescent dye) was oxidized to resorufin (fluorescent pink) wherever the wells contained viable bacterial cells. Thus, the concentration in the last well on each raw that remained blue was considered to entirely inhibit bacterial growth, the MIC. The results are presented in Table S1.

### 2.6. Rheological Analyses

The flow behavior of the biofilm solutions was executed with a modular compact rheometer Anton Paar MCR 72 (Anton Paar, Graz, Austria), equipped with a Peltier plate-plate system (P-PTD 200/Air) with temperature control (temperature range 5 °C to 150 °C). Each sample (~3 mL) was positioned between the two plates, the upper plate with a smooth parallel plate geometry, and 50 mm diameter, and the lower one with a preset temperature at 25 °C and at a gap of 1 mm [42,43,44]. Sample surplus was eliminated prior to measurement, and samples were left 5 min at rest to provide thermal equilibrium preceding the measurements. Measurements were carried out in duplicate with a linearly increasing shear rate from 5 to 300 1/s.

### 2.7. Biofilm Characterization

#### 2.7.1. Physical Measurements

Thickness (µm) was measured with a digital caliper in mm (Lumytools LT15240, Suceava, Romania) at five random points for each film. The measurement was run in duplicate. The final thickness was calculated as the mean value of all measurements (*n* = 10).

Diameter (mm) of each sample was measured with above-mentioned digital caliper at three random points. The measurement was run in duplicate and density values were established as the mean values of all measurements (*n* = 6).

Mass (g) of solid films were weighed with an analytical scale (Kern ALJ 220-5DNM, Germany) with a precision of 0.0001 g. The final weight was the mean value of two measurements.

Density (g/cm^3^) of solid films was calculated according to [21]. The weight of each sample was divided by the surface and thickness, using the following equation:

Density (g/cm^3^) *= w*/*a·t* (1)

where *w* is the weight of sample (g), *a* is the area of sample (cm^2^), *t* is the thickness of the sample (cm). The final thickness was calculated as the mean value of two measurements.

#### 2.7.2. Water Vapor Transmission Rate

Water vapor transmission rate (WVTR) was determined according to the ASTM E96/E96M-10 standard. Films were sealed onto permeability cups (VF2200, TQC Sheen, Molenbaan, The Netherlands) containing approximately 7.5 g CaCl_2_ anhydrous (0% RH). An air gap of 6 mm was kept between CaCl_2_ and film. A total of 10 cm^2^ of film was exposed to water vapor. The cups were then placed into pre-equilibrate desiccator containing a saturated NaCl aqueous solution to 25 ± 1 °C (75% RH) [45]. The cups were weighed 5 times in 24 h. Linear regression was made of the weight gain versus time. *WVTR* was calculated by following equation:

*WVTR* = *S/A* (2)

where *S* is the slope of the straight line (g/h), *A* is the cup mouth area (m^2^), and WVTR is the rate of water vapor transmission (g/h·m^2^). WVTR was conducted in duplicate.

#### 2.7.3. Water Solubility Test

The obtained biofilms were also subjected to a water solubility test at different temperatures, a protocol adapted after Maizura et al., 2007 [46] and Saputri et al., 2018 [47], with slight modifications. Samples of 2 × 2 cm were cut from each biofilm, dried in a desiccator filled with anhydrous calcium chloride, and maintained for 5 days. Biofilm pieces (B_0_) and Whatman paper no. 1 (W_0_) having a diameter of 14.5 cm were weighed to the nearest 0.0001 g. The samples were put in beakers containing 50 mL of double-distilled water and stirred at 300 rpm for 30 min at room temperature (25 °C) and at 60 °C. The biofilms solutions were then filtered through the Whatman papers and dried at 50 °C for 24 h. The filter papers were cooled in the desiccator and weighed (W_1_) again. Samples were measured in duplicates, and the percentage of total soluble matter expressed as solubility (%) was calculated by using the following equation:

Solubility (%) = [B_0_ − (W_1_ − W_0_)]/B_0_ × 100 (3)

where B_0_—weight of biofilm pieces (in grams), W_1_—weight of Whatman filter papers before drying (in grams), W_1_—weight of Whatman filter papers after drying (in grams).

### 2.8. Statistical Analyses

Statistical analysis was performed in Microsoft Excel 2016 and all experiments were run in duplicate (*n* = 2). Results are expressed as mean value ± standard deviation (SD). One-way ANOVA with post hoc Tukey test was used to determine if there were significant differences between Control films (PVA+Gly and PEC+Gly) and films with addition of active compounds (IA, OE, PE) regarding their diameter, mass, thickness, density, water solubility, and WVTR, respectively. Tests were applied for each batch of film (PVA and PEC) separately. For diameter and thickness (*n* = 6 and *n* = 10), tests were applied after descriptive statistical analysis. Skewness values were considered for normal distribution and variance values for homogeneity.

For WVTR, a *t*-test assuming unequal variances was used to determine significant differences between both PVA and PEC when Gly, Gly+IA, Gly+IA+OE, and Gly+IA+PE were incorporated in each matrix. Bifactorial ANOVA with replication was applied to determine if matrix type (PVA and PEC), addition of active compounds (IA, OE, PE), and interaction between them influence water solubility to 25 °C and 60 °C, respectively.

For OE and PE, a *t*-test assuming unequal variances was used to determine the significant differences between the content of each organic acid and phenolic acid extracted from frozen apples and lyophilized apples. A *t*-test assuming unequal variances was also used to determine significant differences for antioxidant activity of OE and PE from frozen apples and lyophilized apples, as well as of OE and PE from frozen apples, and OE and PE from lyophilized apples. All tests were performed at the confidence level of 95%. The symbols used were as follows: ** *p* < 0.01, * *p* < 0.05, ^NS^ *p* > 0.05.

## 3. Results and Discussion

### 3.1. Phenolic and Organic Profile of Apple Pomace Extracts

The AP was used as an organic substrate for the isolation of the organic acids and phenolic compounds. The Red Delicious apple type was analyzed frozen and in lyophilized form, both for organic acid content and for the individual phenolic composition, as presented in Table 1 and Table 2, respectively.

In the case of organic acids profile, malic, citric, succinic, and fumaric acids were identified, and the lyophilization process proved significantly higher efficiency in preserving the content of the total organic acids towards freezing. That is to say, the elevated content of malic, citric, and succinic acids found in the lyophilized fraction might influence the polymerization process and the formation of strong hydrogen bonds in connection with the base matrices, both in PVA and pectin-based films [48]. As shown by the scientific literature [49,50], the presence of organic acids in PVA and pectin-based solutions facilitates cross-linking, resulting in improved mechanical properties of the coating material.

Besides being efficient cross-linkers in biopolymer packaging, organic acids have high antioxidant activity (as observed in Section 3.2.1), and they can efficiently hinder the cumulation of reactive oxygen species—ROS (O_2_^−^, H_2_O_2_) in freshly harvested fruits. Thus, their integration in active packaging might alter the modifications in pH or even the action of oxygen-related enzymes and maintain the antioxidant activity in fruits or vegetables during storage [51]. The use of organic acids is intensively studied as they have proved to increase the shelf-life of several foodstuffs, such as bread [43], various fermented foods [52], and freshly collected crops, by inhibiting fruit-browning, and altogether improving their quality and slowing the decline in their overall characteristics [53].

Regarding the profile of total phenolic compounds, such as for organic acids, the lyophilization process was proved to maintain the highest values towards the freezing of AP samples (Table 3). Among the phenolic subclasses identified in the Red Delicious apple type, the most abundant were hydroxycinnamic acids, flavanols, and dihydrochalcones that were present both in frozen and in lyophilized forms, with the highest amount recorded for chlorogenic acid in lyophilized AP (1173.13 ± 0.19 µg/g). Phenolic compounds such as chlorogenic acid, epicatechin, or procyanidins imprint good mechanical properties to the end product in terms of firmness, elongation breaking point, tensile strength, and even low oxygen permeability [54,55]. In addition, several studies pointed out the antioxidant and antimicrobial properties of bioactive edible coating materials enriched with polyphenols such as chlorogenic acid, procyanidins, or isoquercitrin [56,57,58].

Among the flavonols group, the highest concentration of the seven identified phenols was quercetin-glucoside (Isoquercitrin) at 217.84 ± 0.08 μg/g in frozen AP, and epicatechin 736.21 ± 0.44 μg/g in the lyophilized AP powder. Quercetin-glucoside, besides quercetin-rutinoside (Rutin), quercetin-arabinoside (Avicularin), quercetin-(malonyl)-glucoside, and quercetin-rhamnoside (Quercitrin), are derivatives of quercetin, and are polyhydroxy compound bioflavonoids. In addition to the quercetin derivatives, other phenols were also identified, such as epicatechin and procyanidin dimmer, both with a significant increase (*p* < 0.01) after the lyophilization process of 17.3% (723.07 ± 0.06 μg/g), and 25.86% (736.21 ± 0.44 μg/g). Based on Ezati and Rhim, they possess powerful antimicrobial and antioxidant agents and can effectively protect against UV by diminishing the transparency of biofilms [59].

Three hydroxycinnamic acids were identified 5-Caffeoylquinic acid (Chlorogenic acid) with the highest content in the lyophilized form of 1173.13 ± 0.19 μg/g, and with a substantial increase of 20.77% and 20.42% of caffeic acid-glucoside, and p-coumaroylquinic acid, respectively. Although they are easily oxidized and unstable at increased temperature, through lyophilization, there was only an increase in their concentration. These compounds can be found in apples and also in berries, prunes, pear and, especially in coffee, and they also possess high antioxidant activity, and anti-viral, inflammatory and microbial activity [60]. Among other discovered phenols (Gallic acid-glucoside, Cyanidin-glucoside, and Phloretin-xylosyl-glucoside), an essential compound especially found in apple is phloretin-glucoside (Phloridzin), belonging to dihydrochalcone glycosides, which possesses tumor growth inhibitory effects. In this case, the lyophilization process also increased the concentration by 15.85%, to a final value of 720.39 ± 0.09 μg/g.

Consequently, several organic and phenolic compounds can be found in the by-products of AP, which possess multiple advantages to health, such as enhanced oxidative stress-related diseases, diabetes, obesity, and others [61,62,63].

### 3.2. Evaluation of Apple Pomace Extracts

#### 3.2.1. Antioxidant Activity of Extracts before and after Lyophilization

The modifications in antioxidant activity of the organic and phenolic extracts from AP were evaluated by the DPPH free radical scavenging capacity, and the obtained results are displayed in Table 3. After lyophilization, the antioxidant activity of the phenolic extracts presented a statistically significant growth of 14.4% (*p* < 0.01) and the organic extracts of 47.16% (*p* < 0.01).

Regarding the bioactive potential of AP, several similar studies presented increased results after lyophilization [64,65] or even after the thermal treatment of these by-products [66]. The antioxidant activity is highly influenced by the drying and processing of AP and the extraction method, as seen in our results. In our case, the organic extraction presented the best results with a final value of 166.69 ± 0.47 μM Trolox/100 g dry weight, which suggests that these samples contained a higher quantity of polyphenols.

The high antioxidant activity observed in the organic extracts can be attributed to the fact that they have acidic properties and are identified as weak acids. Citric acid (2-hydroxy-1,2,3-propane tricarboxylic acid) is an acknowledged antioxidant, especially in oils and fats, due to the large number of metallic salts present in its structure comprising iron, magnesium, copper, calcium, and manganese [52]. All four identified organic acids (malic, citric, fumaric, and succinic acids) are part of the Krebs cycle, and all of them present important health-related effects, and are primarily used as food antioxidants. Besides having antioxidant properties, they also act as flavoring, acidulant, buffering, and chelating agents [67].

#### 3.2.2. Antimicrobial Activity of Extracts and Biofilms before and after Lyophilization

The antibacterial activity of AP phenolic and organic extracts is presented in Supplementary Table S1. As can be seen, none of the extracts or biofilm solutions enriched with extracts presented any antimicrobial activity against the selected strains. This aspect may be due to the fact that the content of the organic acids and phenolic compounds was lower towards the minimum inhibitory concentration against the tested pathogens. As is shown in the literature, the most used organic acids that exert strong antimicrobial activity are lactic, sorbic, benzoic, and propionic acids. Their antimicrobial activity is interconnected with their undissociated form, polarity, and lipophilic nature [52].

### 3.3. Rheological Measurements of Biofilm Solutions

The effect of IA and the integration of phenolic and organic acid extracts in the biofilm solutions have also been evaluated through the film-forming solution’s viscosity measurements. These rheological studies display the deformation of the sample due to the influence of mechanical stresses and allow the examination of the final characteristics that help in the optimization of the manufacturing process.

**Figure 1:**
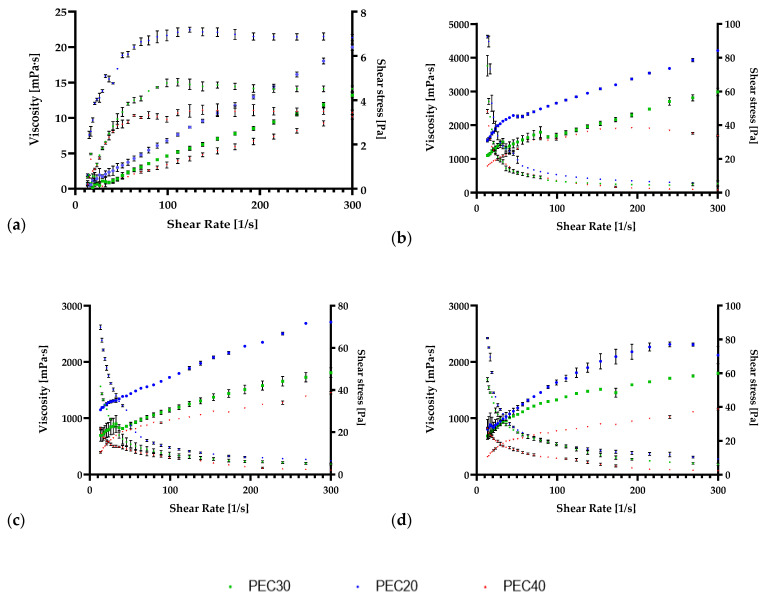
Shear viscosity and shear rate of pectin and itaconic acid-based biofilms at 20, 30, and 40 °C temperature. The reported mean values with standard deviation (±) are displayed for (**a**) pectin + glycerol; (**b**) pectin + glycerol+ itaconic acid; (**c**) pectin + glycerol+ itaconic acid + phenolic extract; (**d**) pectin + glycerol + itaconic acid + organic extract.

**Figure 2:**
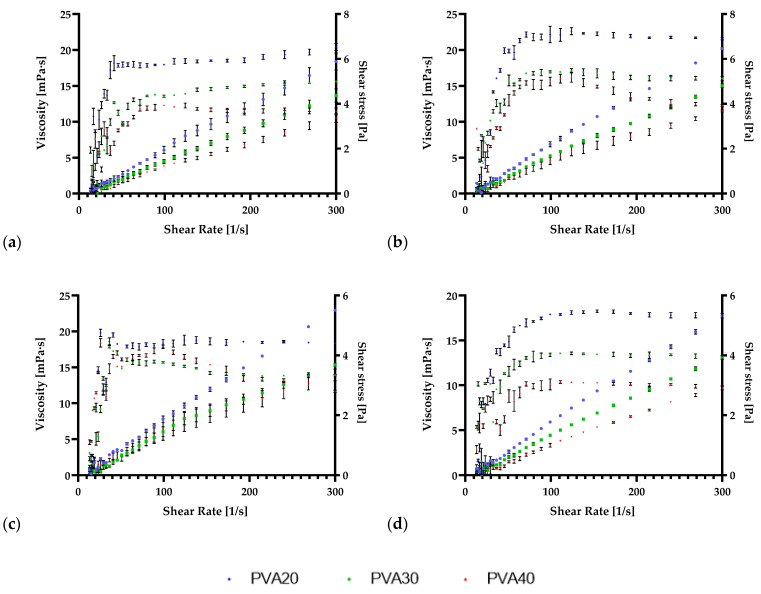
Shear viscosity and shear rate of PVA and itaconic acid-based biofilms at 20, 30, and 40 °C temperature. The reported mean values with standard deviation (±) are displayed for (**a**) PVA + glycerol; (**b**) PVA + glycerol+ itaconic acid; (**c**) PVA + glycerol+ itaconic acid + phenolic extract; (**d**) PVA + glycerol + itaconic acid + organic extract.

These measurements have been analyzed at three different temperatures between 20 and 40°. The viscosity and shear stress of the solutions were measured for a shear rate ranging from 5 to 300 1/s. Both control biofilms based on PVA or pectin presented a shear-thickening (dilatant) behavior when only glycerol or, in the case of pectin glycerol and CaCl_2_ were added, as can be observed in Figure 1 and Figure 2a. However, after the integration of IA together with pectin, all the solutions displayed a shear-thinning (pseudo-plastic) behavior as can be observed in Figure 1 and Figure 2b–d. Regarding PVA, the integration of IA did not alter the behavior of the film-forming solutions.

Pectin is a polysaccharide delineated primarily of an α-1,4-D-galacturonic acid residue and constructed from at least 17 distinct monosaccharides [68]. In this case, pectin was extracted from AP and being hydrophilic, the addition of Ca^2+^ ions was selected to achieve an acidic state that promotes gelification [69]. With the integration of IA macromolecule and glycerol polyol, the physicochemical characteristics are improved, as was also suggested by Lewandowska et al. [70].

PVA also encompasses unique and favorable characteristics that promote its use in biodegradable active packaging. Carbon atoms delineate its structure, and the presence of a hydroxyl group is advantageous for forming firm hydrogen bonds among hydroxyl groups [21]. The integration of glycerol, a by-product of the biodiesel industry [71], is promoted, especially because it improves the physical characteristics of the formed biofilms, as it was also proved in recent studies [20,21]. The formation of intra- and intermolecular hydrogen bonds also has an impact on the rheological features of the biofilm film-forming solutions [72]. The temperature alteration also affects the viscosity, with the increase in temperature, the viscosity decreases.

The highest value for viscosity was observed with the integration of IA in the biopolymer solution, reaching values of 22.6 ± 0.41 mPa·s at 20 °C. In the case of pectin, the integration of IA produced an outset of 4627 ± 33.40 mPa·s, at 20 °C and a final viscosity of 280.8 ± 0.49 mPa·s at 20 °C. Nevertheless, with the temperature growth, the viscosity decreased in both PVA and pectin biofilms and in the case of IA integration. The addition of phenolic and organic extracts diminished biofilm solution viscosities, which was also observed in similar studies [20,21,43].

### 3.4. Biofilm Characterization

**Figure 3:**
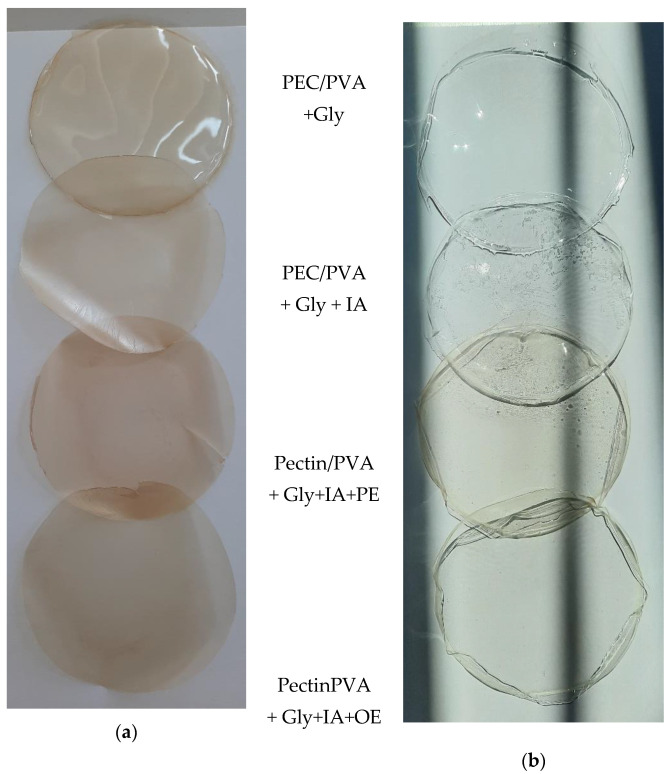
The physical aspect of the solid films with (**a**) pectin, and (**b**) PVA based on itaconic acid and extract integration.

The physical aspect of the solid biofilms with pectin or PVA, based on IA and enriched with phenolic and organic extracts, is presented in Figure 3a–b. Both biopolymers are biocompatible and considered safe for human intake by FAO/WHO, and are highly studied in the field of food and beverage manufacturing and packaging, as drug carriers, in tissue engineering, and as wound covering materials in the pharmaceutical sector [73].

Biofilms with PVA were transparent, and the integration of IA did not affect the biofilm’s transparency or color. In the case of PVA, only the phenolic extract gave a slightly yellowish color. Pectin films were orange, but without IA, they were also transparent. The integration of IA gave an opacity to biofilms. In addition, the integration of the phenolic extract gave a more intense color. Although transparent films are more appreciated, the positive effect of opaque packaging on light’s degradative effect is constantly increasing [74].

#### 3.4.1. Thickness, Diameter, Mass, and Density

Eight types of biofilms were attained in the current study, represented as PVA/pectin (PEC)+Gly, enriched with IA and afterwards also enriched with organic (OE) or phenolic (PE) extract, as presented in Table 4. The amount of 15 mL was sufficient for the diameter of a Petri dish, and after solidification at room temperature for approx. 48 h, they were easily removed from the plates. The pectin-based biofilm was more fragile than the PVA-based, especially after the integration of IA, but all of them were homogeneous. Glycerol was incorporated to offer more elasticity, and the phenolic extract also enchased a more intense color. The enrichment with phenolic/organic extract increased the diameter of the biofilms, while IA reduced its diameter compared to the control (PVA+Gly), causing them to be more compact.

The thickness of PVA biofilms decreased significantly (*p* < 0.05) from 92 ± 23 µm to 74 ± 10 µm with extract integration, while in case of pectin biofilms, the thickness increased (48 ± 17 µm) significantly (*p* < 0.01), especially with organic extract enrichment (75 ± 24 µm). The mass of biofilms increased with the incorporation of IA and extracts. At the same time, the density was the highest in PEC+Gly+IA+PE biofilm with 1.63 ± 0.14 g·cm^−3^, and the lowest in PEC+Gly+IA+OE with a final density of 1.05 ± 0.21 g·cm^−3^.

#### 3.4.2. Water Vapor Transmission Rate

The water barrier properties of PVA and pectin films are essential in predicting the packaged product’s shelf-life and applicability. A lower WVTR is highly desirable to prevent moisture transfer between food and the external environment. The lower the WVTR, the higher the moisture barrier. A high moisture barrier can increase the quality of food products and extend their shelf-life [75].

**Figure 4:**
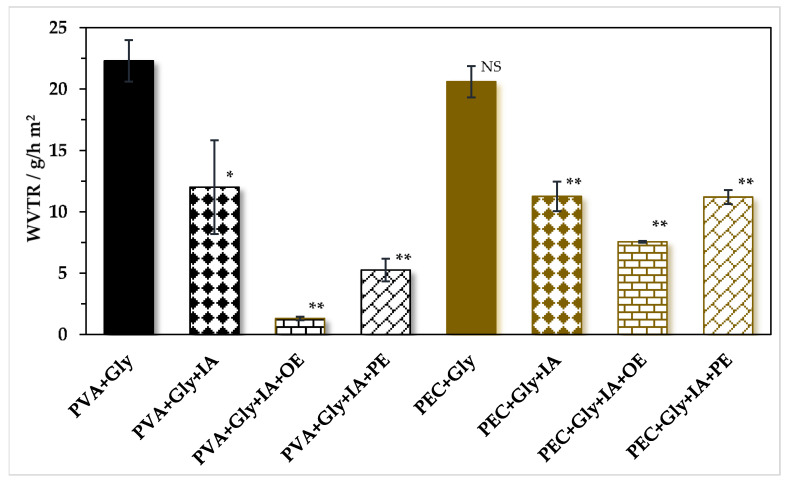
Water vapor transmission rate (WVTR) of films. Mean ± SD (*n* = 2). * *p* < 0.05, ** *p* < 0.01, ^NS^ *p* > 0.05 express significant differences in comparison to PVA+Gly and PEC+Gly, respectively. PVA-PolyVinyl Alcohol, IA-Itaconic Acid, Gly—glycerol, OE—organic Extract, PE—Phenolic Extract.

PVA and pectin contain hydroxylic groups (−OH) that are strongly hydrophilic. Due to their high hydrophilicity, the moisture barrier of PVA and pectin films is very low. Therefore, in this study, WVTR had the highest values for PVA+Gly (22.30 ± 1.70 g/h·m^2^) and pectin+Gly (20.60 ± 1.27 g/h·m^2^), respectively (Figure 4). When IA was added in both PVA (*p* < 0.05) and pectin (*p* < 0.01) films, WVTR significantly decreased to 12 ± 3.82 g/h·m^2^ and 11.25 ± 1.20 g/h·m^2^. IA contains two carboxylic (-COOH) groups [13]. The COOH groups of IA interact with the -OH groups (esterification reaction), leading to the liberation of one molecule of water (H_2_O) and the formation of one less hydrophilic carbonyl group (-C=O) for each –COOH. Therefore, the cross-linking of IA to both PVA and pectin matrices leads to more compact films (due to eliminated H_2_O molecules) and less hydrophilic films (due to the formed –C=O groups), which may explain the lower WVTR values. Furthermore, when OE and PE were added, WVTR significantly decreased in PVA and pectin films (*p* < 0.01). OE reduced WVTR by 94% (1.30 ± 0.14 g/h·m^2^) for PVA films and by 63% (7.55 ± 0.07 g/h·m^2^) for pectin films. OE was the most effective active compound for reducing WVTR of PVA and pectin films in this study.

Organic acids also contain -COOH that acts as strong cross-linkers and reduce the availability of free -OH groups in the PVA and PEC structures. For example, WVTR was reduced when citric acid was added to chitosan films [53]. The same effect was observed when hydroxy citric acid was added to chitosan/guar gum/PVA films. The cross-linking of hydroxy citric acid increased the hydrophobic nature of the composite film [76]. According to HPLC analysis, citric acid was found in OE used in this study and may influence WVTR of samples. Phenolic acids contain also -COOH groups. However, phenolic acids found in PE used in this study have -OH groups (e.g., gallic acid, rutin, caffeic acid) attached to their phenolic ring. This can explain no significant differences (*p* < 0.05) between WVTR values obtained for addition of IA with or without PE in both PVA and pectin films. In conclusion, addition of IA with or without apple extracts can significantly reduce WVTR in both PVA and pectin matrices. Incorporation of OE had the highest effect to reduce WVTR of PVA (by 94%) and pectin (by 63%) films.

Furthermore, WVTR of PVA and PEC films incorporating apple extracts was lower than WVTR of pure low-density polyethylene (LDPE) films (371.18 ± 20.54 g/d·m^2^). LDPE films were recently developed by melt extrusion and hot pressing [77]. Thus, the obtained films incorporating active compounds may have an even better moisture barrier than conventional plastic films.

#### 3.4.3. Water Solubility of the Biofilms

PVA and pectin are among the most used biodegradable polymers highly rich in hydroxyl groups, units that give increased water solubility to the whole structure of the polymers [19,78]. IA is another water-soluble compound largely exploited as a co-monomer in constructing different industrial polymers or in designing various coating materials [13]. In connection with other polymeric structures such as PVA or pectin, IA increases the physical resistance at the break, elasticity, or stability of the end products, imprinting them, at the same time, higher resistance to water activity [79].

**Figure 5:**
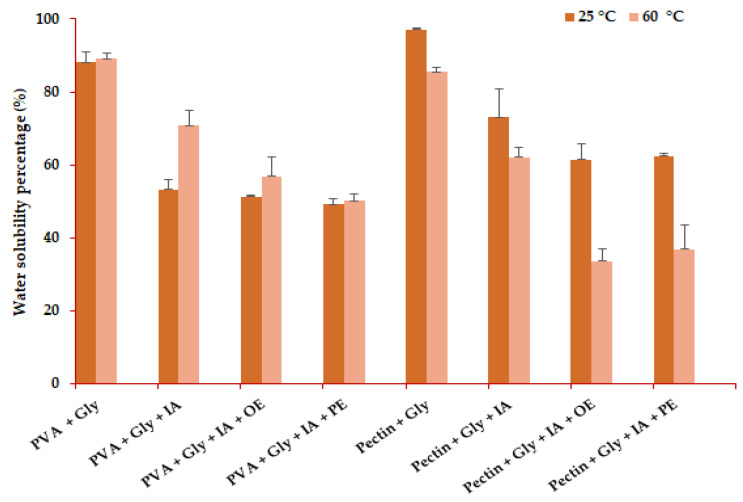
The results obtained for water solubility of the PVA and pectin-based biofilms, enriched with IA, organic extract and phenolic extract. PVA-PolyVinyl Alcohol, IA-Itaconic Acid, Gly—glycerol, OE—organic Extract, PE—Phenolic Extract. The results are expressed as mean values (±SD).

From the current study, during the sample dissolving process, it was observed that the biofilms containing IA have swollen by increasing their surfaces before breaking and dissolving. Moreover, as in the case of the present work, adding IA to the PVA and pectin-based solutions resulted in decreased solubility in the water of the obtained biofilms, as can be observed from Figure 5.

The solubility test represents the physical-chemical exam for samples to resist water activity. It also brings valuable information about the polymeric structures or the coating matrices in close connection with their possible applications [80]. For this work, the 2 × 2 cm biofilm pieces were sunk in double-distilled water, stirred for 30 min at 25 °C and 60 °C, and then filtered through Whatman papers no. 1. All the samples have been completely or partially dissolved in water. Most of the PVA-based samples passed easily through the filter papers (less than 30 min), while the pectin-based biofilms took longer to be filtered entirely (up to 2 h and 30 min). Water solubility was significantly (*p* < 0.01) affected by the film matrix base (PVA and pectin) and by the addition of active compounds at both 25 °C and 60 °C. Moreover, the water solubility is significantly affected by the interaction between the film matrix and active compounds (IA, organic extract, phenolic extract) in the case of dissolution at 60 °C (*p* < 0.05).

The increase in temperature positively influenced the samples’ dissolution and the filtration time in the case of PVA films. In contrast, the solubility percentage for pectin-based films was higher at room temperature, as seen in Figure 5. PVA-based films proved high water solubility, both at room temperature and above (up to 88.10 ± 2.91% and 89.06 ± 1.50% for PVA mixed with glycerol and dissolved at 25 °C and 60 °C). Still, the addition of IA, organic extract, and phenolic extract based on apple have significantly (*p* < 0.05) diminished the water solubility of the PVA biofilms, both at 25 °C and 60 °C. This aspect can be attributed to forming strong hydrogen bonds between PVA hydroxyl units and the analogous groups [13]. Moreover, for PVA+Gly+IA samples, no significant (*p* > 0.05) influence on water solubility at 60 °C by comparison with PVA+Gly was observed. By comparing our results for the solubility percentage of PVA-based samples, we found that these are similar to those reported by Oyeoka et al. [81]. They achieved a water solubility of 71% for biofilms consisting of PVA 5% and gelatin 5%. The same authors reported lower water solubility, namely 63% and 60%, for the same PVA-gelatin films reinforced with 5% and 10% crystalline nanocellulose [81]. In another study, the authors achieved a water solubility percentage of 75% for the PVA-chitosan matrix (2% PVA, 1.5% chitosan) acidified with 1% acetic acid, for a mixture obtained at room temperature overnight [80]. As the polymeric blends form strong bonds among the hydroxyl units, the more decreased is their solubility, a fact evidenced by Abdullah and Dong [82], who observed that 5% PVA-based films plasticized with glycerol and supplemented with starch and a halloysite nanotube have a decreased water solubility percentage of nearly 40%, at a temperature of 35 °C [82].

In the case of pectin-based biofilms, these were slightly more rigid compared with the PVA-based ones and with facile dissolution in water. It was observed that the highest water solubility percentage (97.07 ± 0.33%) was recorded at 25 °C for pectin-based samples plasticized with glycerol, but with no addition of other compounds. The addition of IA, organic extract, and phenols brought higher resistance and lower water solubility to the biofilm samples, as it was obtained a water solubility percentage of 72.98 ± 7.91% (pectin+Gly+IA), 61.37 ± 4.33% (pectin+Gly+IA+OE), 62.43 ± 0.68% (pectin+Gly+IA+PE). That is to say, the addition of organic and phenolic extracts significantly (*p* < 0.05) influences the water solubility at 60 °C in both PVA and pectin-based films in comparison to the addition of IA without extracts. At 60 °C, even though the filtration rate was improved towards that achieved at 25 °C, the total soluble matter was lower at elevated temperature, as it was obtained: 85.58 ± 1.05% (pectin+Gly), 62.14 ± 2.71% (pectin+Gly+IA), 33.54 ± 3.49% (pectin+Gly+IA+OE), 36.84 ± 6.61% (pectin+Gly+IA+PE). Surprisingly, the addition of organic and phenolic extracts to pectin+Gly+IA biofilms gave firmness and resistance against water activity to the initial polymeric matrix. This effect can be attributed to the presence of other organic acids besides IA, such as fumaric, succinic, malic, and citric acids, and phenols such as flavonols, hydroxybenzoic and hydroxycinnamic acids that have the capacity to strengthen the cross-linking among the bioactive molecules and to generate more hydrogen bonds between polysaccharides by giving improved mechanical properties to the entire structure. The results reported for trials based on pectin are comparable with those shown in the scientific literature. For example, Guadarrama-Lezama et al. [83] obtained a coating material with improved thermal and mechanical properties by using a pectin-based solution supplemented with nopal mucilage, citric acid, and glycerol. The authors observed that the addition of different concentrations of nopal mucilage (up to 20%) to a pectin solution of 2% decreases gradually the solubility in water of the obtained coating material, from over 80% (with no addition of nopal mucilage) to less than 60% (with 20% addition of nopal mucilage). Anyhow, the authors suggested that up to 12% mucilage supplementation is optimal for achieving a coating material with good mechanical and thermal properties, recording a water solubility percentage over 75% for this situation [83]. Another study pointed out that pH values considerably influence the solubility in water of pectin-based coatings. Giancone et al. [84] observed that high methoxyl pectin-based edible films show high solubility in water (99.0  ±  0.6% at a density of 2.5 mg/cm^2^) at low pH (around 4). At a pH value of 4, the water solubility percentage decreases to 83  ±  5.0% with the increase of density to 5.8 mg/cm^2^_._ When the pH values reach 8, the solubility percentage is around 78% (at a density of 2.5 mg/cm^2^) and decreases to 53  ±  5.0% with the increase of density (5.8 mg/cm^2^) [84].

PVA and pectin-based biofilms are easily dissolvable in water. Still, incorporating other bioactive structures such as IA or natural extracts based on various agro-industrial products, edible coating materials can be obtained with increased mechanical and physical properties and high resistance to water activity.

## 4. Conclusions

Both biopolymers, pectin and PVA, are highly used in the food and pharmaceutical sector. Due to their efficient cross-linking characteristics with other materials, such as IA, they can be efficiently used as biopolymers. The supplementation of PVA and pectin-based matrices with IA and with OE and PE derived from AP positively impacted the rheological properties of the film-forming solutions. The bioactive compounds derived from these extracts were increased after lyophilization, together with enhancement of their antioxidant activity. In addition, it also positively influenced the physical-chemical characteristics of the solid films in terms of antioxidant potential and mechanical resistance given by the low water vapor permeability along with decreased solubility in water.

The biofilms constructed on PVA and pectin matrices gained resistance against intrinsic and extrinsic factors by adding organic acids and phenolic compounds. Still, more research is necessary to identify the optimal applications for the proposed formulations, along with biodegradability tests (in water, and soil). Knowledge acquired from this experiment will be an essential precursor for the practical application of AP that can be suitable for bio-composite packaging materials.

## Figures and Tables

**Table 1:** Content of organic acids in AP samples, expressed in mg/g.

Apple Treatment (mg/g)/Organic Acid	Malic Acid	Citric Acid	Succinic Acid	Fumaric Acid	Total
Frozen	2.671 ± 0.076 **	0.840 ± 0.005 *	0.085 ± 0.006 *	0.001 ± 0.000 *	3.597 ± 0.066 **
Lyophilized	14.730 ± 0.124	4.556 ± 0.109	0.530 ± 0.023	0.012 ± 0.001	19.828 ± 0.211

Mean ± SD (*n* = 2); ** *p* < 0.01 and * *p* < 0.05. Frozen values in comparison to lyophilized values for each organic acid.

**Table 2:** Individual phenolic composition of the AP samples, expressed in μg/g.

Peak No.	R_t_ (min)	UV λ_max_ (nm)	[M+H]^+^ (*m*/*z*)	Compound	Subclass	Frozen	Lyophilized
1	3.25	280	333	Gallic acid-glucoside	Hydroxybenzoic acid	45.47 ± 0.07 **	106.62 ± 0.14
2	10.70	280, 520	449, 287	Cyanidin-glucoside	Anthocyanin	20.29 ± 0.00 **	78.47 ± 0.18
3	12.45	323	355	5-Caffeoylquinic acid (Chlorogenic acid)	Hydroxycinnamic acid	156.71 ± 0.09 **	1173.13 ± 0.19
4	13.09	280	579, 291	Procyanidin dimmer	Flavanol	124.70 ± 0.10 **	723.07 ± 0.06
5	13.62	322	343	Caffeic acid-glucoside	Hydroxycinnamic acid	42.10 ± 0.03 **	202.74 ± 0.29
6	13.74	280	291	Epicatechin	Flavanol	190.36 ± 0.04 **	736.21 ± 0.44
7	14.23	316	339	*p*-Coumaroylquinic acid	Hydroxycinnamic acid	45.41 ± 0.01 **	222.40 ± 0.04
8	15.53	259, 348	611, *303*	Quercetin-rutinoside (Rutin)	Flavonol	11.85 ± 0.16 **	34.66 ± 0.07
9	16.06	259, 350	465, *303*	Quercetin-glucoside (Isoquercitrin)	Flavonol	217.84 ± 0.08 **	358.58 ± 0.07
10	16.73	260, 348	435, *303*	Quercetin-arabinoside (Avicularin)	Flavonol	79.68 ± 0.23 **	11.58 ± 0.09
11	17.01	285	569, *275*	Phloretin-xylosyl-glucoside	Dihydrochalcone	34.61 ± 0.09 **	201.59 ± 0.09
12	17.21	260, 349	551, *303*	Quercetin-(malonyl)-glucoside	Flavonol	129.17 ± 0.09 **	118.88 ± 0.04
13	17.39	260, 347	449, *303*	Quercetin-rhamnoside (Quercitrin)	Flavonol	88.46 ± 0.06 **	164.07 ± 0.09
14	18.35	287	437, *275*	Phloretin-glucoside (Phloridzin)	Dihydrochalcone	114.18 ± 0.03 **	720.39 ± 0.09
				Total Phenolics		1300.82 ± 0.10 **	4952.40 ± 0.08

Rt—Retention time; Mean ± SD (*n* = 2); ** *p* < 0.01. Frozen values in comparison to lyophilized values for each phenolic acid.

**Table 3:** Antioxidant activity of apple pomace extracts before and after lyophilization.

1-AP-PE	9.70 ± 0.078 **	μM Trolox/100 g fresh Weight
2-Lyophilized AP-PE	67.45 ± 0.28 **	μM Trolox/100 g dry weight
3-AP-OE	78.61 ± 0.24 **	μM Trolox/100 g fresh weight
4-Lyophilized AP-OE	166.69 ± 0.47 **	μM Trolox/100 g dry weight

OE—organic extract; PE—phenolic extract; AP—apple pomace; Mean ± SD (*n* = 2); ** *p* < 0.01.

**Table 4:** Thickness, diameter, mass, and density of films.

Films	Thickness/µm	Diameter/mm	Mass/g	Density/g·cm^−3^
PVA+Gly	92 ± 23	53.54 ± 0.29	0.19 ± 0.00	1.30 ± 0.16
PVA+Gly+IA	84 ± 8 ^NS^	53.40 ± 0.03 ^NS^	0.24 ± 0.01 **	1.55 ± 0.10 ^NS^
PVA+Gly+IA+OE	74 ± 10 *	53.87 ± 0.29 ^NS^	0.22 ± 0.01 *	1.33 ± 0.02 *
PVA+Gly+IA+PE	74 ± 8 *	53.95 ± 0.39 ^NS^	0.26 ± 0.01 **	1.55 ± 0.10 **
PEC+ Gly	48 ± 17	53.35 ± 0.13	0.12 ± 0.01	1.21 ± 0.29
PEC+Gly+IA	53 ± 5 ^NS^	53.22 ± 0.22 ^NS^	0.17 ± 0.00 ^NS^	1.46 ± 0.05 ^NS^
PEC+Gly+IA+OE	75 ± 24 **	53.87 ± 0.29 **	0.17 ± 0.01 ^NS^	1.05 ± 0.21 ^NS^
PEC+Gly+IA+PE	55 ± 5 ^NS^	53.39 ± 0.25 ^NS^	0.20 ± 0.02 *	1.63 ± 0.14 ^NS^

Mean ± SD (thickness, *n* = 10; diameter, *n* = 6; mass, *n* = 2; density, *n* = 2); * *p* < 0.05, ** *p* < 0.01, ^NS^ *p* > 0.05 express significant differences in comparison to PVA+Gly and PEC+Gly, respectively.

## Data Availability

Data is contained within the article.

## Supplementary Materials

The following supporting information can be downloaded at: https://www.mdpi.com/article/10.3390/antiox11091729/s1, Figure S1: Chromatograms of organic acids (a). standard: 1-oxalic Rt = 2.94 min; 2-malic Rt = 3.15 min; 3-citric Rt = 3.31 min; 4-succinic Rt = 3.76 min; 5-fumaric Rt = 4.18 min; (B). Frozen apple; (c). Lyophilized apple; Figure S2. Chromatograms of phenolic compounds (a) Frozen apple—280 nm, (b) Frozen apple—340 nm; (c) Frozen apple–520 nm; (d) Lyophilized apple—280 nm; (e) Lyophilized apple—340 nm; (f) Lyophilized apple—520 nm. Table S1. Minimum inhibitory concentration against *E. coli*, *S. aureus*, *S. epidermidis*, *P. aeruginosa*, *E. faecalis*, *S. pyand* and *S. enterica.*
