# Supplementary material for: Development of Pectin and Poly(vinyl alcohol)-Based Active Packaging Enriched with Itaconic Acid and Apple Pomace-Derived Antioxidants
Source: Antioxidants (Basel). 2022 Aug 31;11(9):1729. doi: 10.3390/antiox11091729 (PMC9495313; doi:10.3390/antiox11091729)
Supplement: Supplementary file 1 [file antioxidants-11-01729-s001.zip › antioxidants-1884125-supplementary.pdf]

Figure S1. Chromatograms of organic acids **(a).** standard: 1-oxalic Rt=2.94min; 2-malic Rt=3.15 min; 3-citric Rt=3.31 min; 4-succinic Rt=3.76 min; 5-fumaric Rt=4.18 min; **(B).** Frozen apple; **(c).** Lyophilized apple

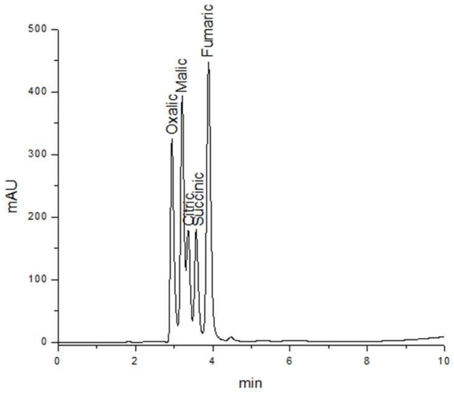

(a)

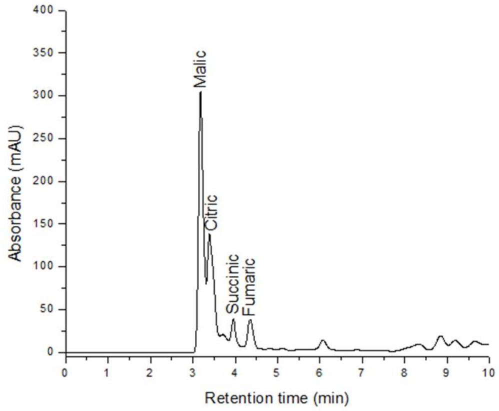

(b)

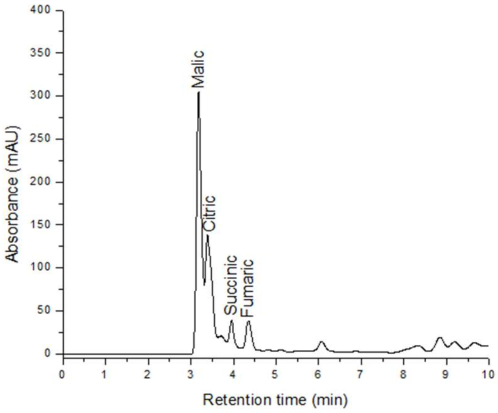

(c)

Figure S2. Chromatograms of phenolic compounds **(a)** Frozen apple - 280 nm, **(b)** Frozen apple - 340 nm; **(c)** Frozen apple - 520 nm; **(d)** Lyophilized apple - 280 nm; **(e)** Lyophilized apple - 340 nm; **(f)** Lyophilized apple - 520 nm

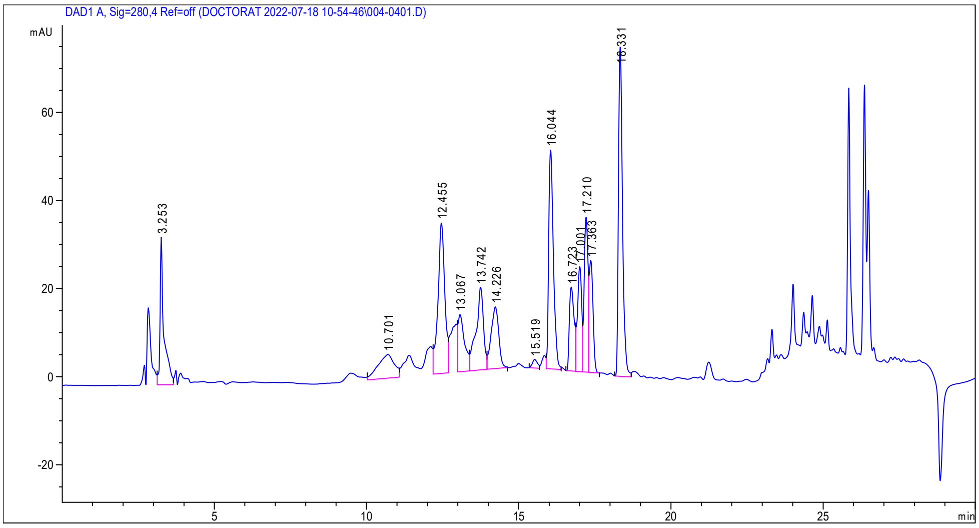

(a)

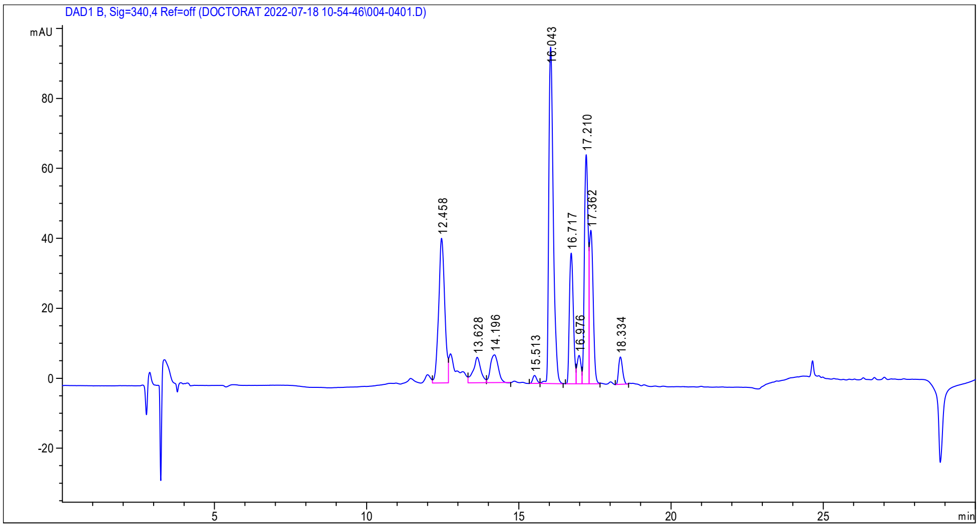

(b)

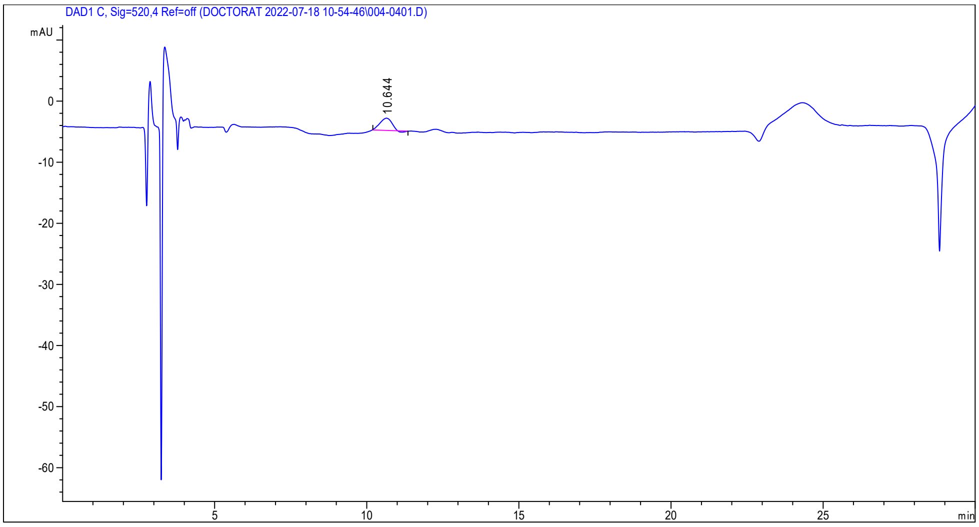
DAD1 C, Sig=520,4 Ref=off (DOCTORAT 2022-07-18 10-54-46\004-0401.D)
mAU
10.644
min
(c)

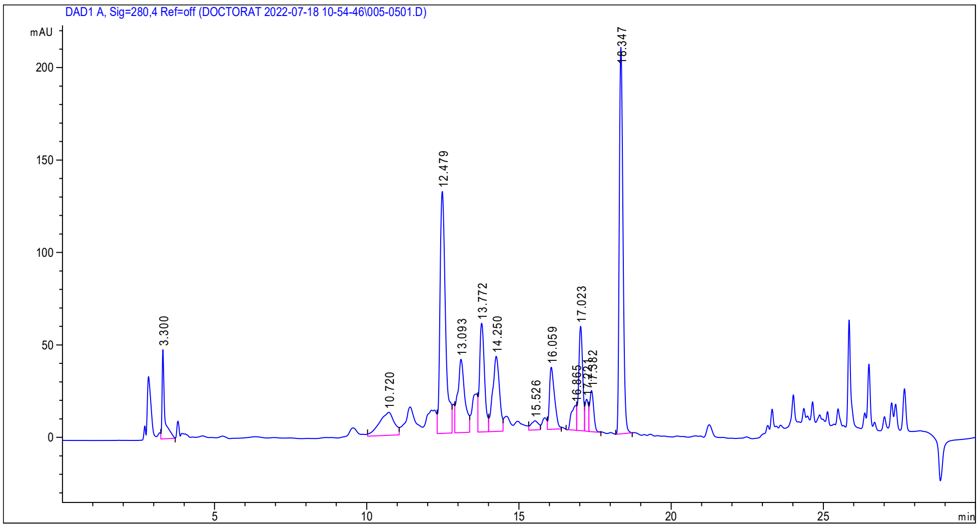
DAD1 A, Sig=280,4 Ref=off (DOCTORAT 2022-07-18 10-54-46\005-0501.D)
mAU
3.300
10.720
12.479
13.093
13.772
14.250
15.526
16.059
16.865
17.023
17.382
18.347
min
(d)

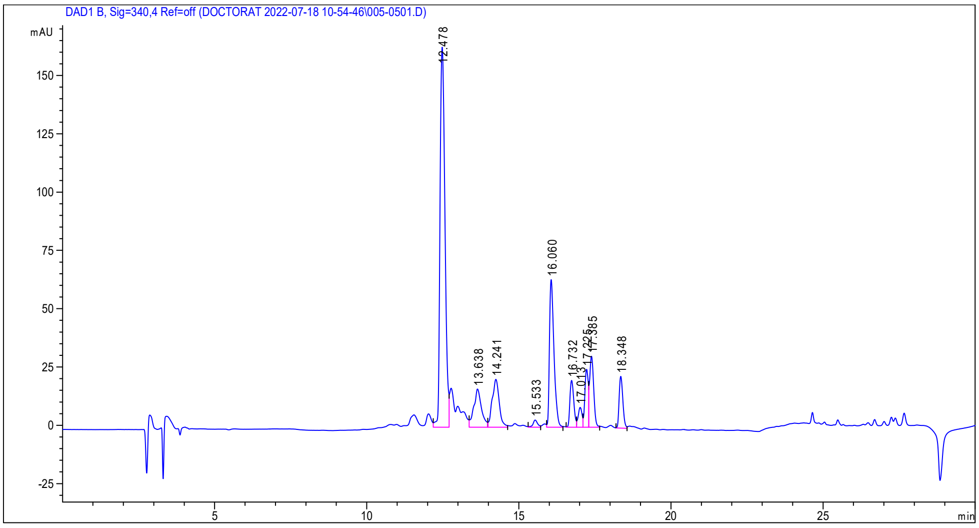
DAD1 B, Sig=340,4 Ref=off (DOCTORAT 2022-07-18 10-54-46\005-0501.D)
mAU
12.478
13.638
14.241
15.533
16.060
16.732
17.385
18.348
min
(e)

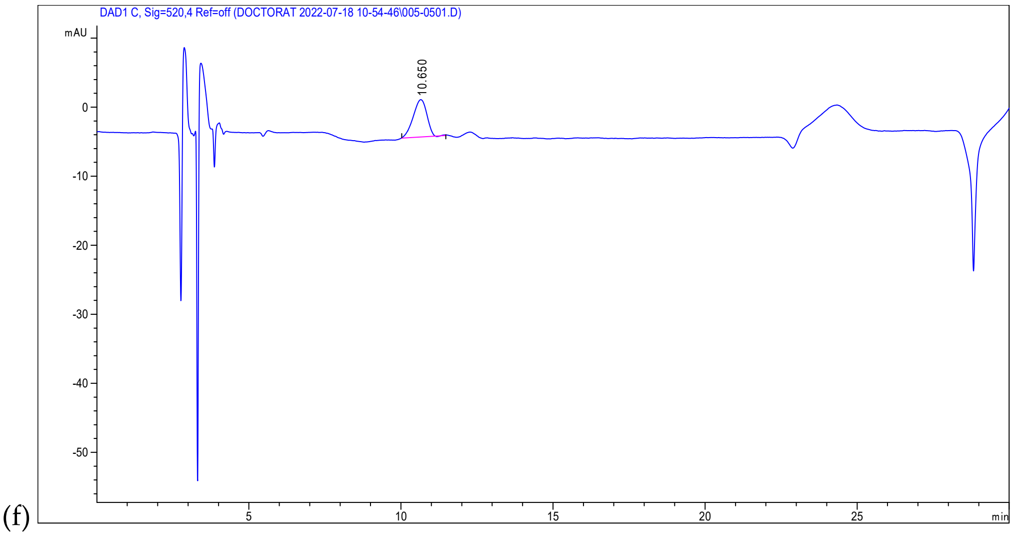
DAD1 C, Sig=520,4 Ref=off (DOCTORAT 2022-07-18 10-54-46\005-0501.D)
mAU
0
-10
-20
-30
-40
-50
10.650
5
10
15
20
25
min
(f)

Table S1. Minimum inhibitory concentration against *E. coli, S. aureus, S. epidermidis, P. aeruginosa, E. faecalis, S. pyand* and *S. enterica.*

	Gram (+) Bacteria					Gram (-) Bacteria	
Samples	*S. aureus*	*S. epidermidis*	*E. coli*	*E. faecalis*	*S. pyogenes*	*P. aeruginosa*	*S. enterica*
AP-PE	n.b	n.b	n.b	n.b.	n.b	n.b	n.b.
FD-AP-PE	n.b	n.b	n.b	n.b.	n.b	n.b	n.b.
AP-OE	n.b	n.b	n.b	n.b.	n.b	n.b	n.b.
FD-AP-OE	n.b	n.b	n.b	n.b.	n.b	n.b	n.b.
B-PE-PVA	n.b	n.b	n.b	n.b.	n.b	n.b	n.b.
B-PE-PEC	n.b	n.b	n.b	n.b.	n.b	n.b	n.b.
B-OE-PVA	n.b	n.b	n.b	n.b.	n.b	n.b	n.b.
B-OE-PEC	n.b	n.b	n.b	n.b.	n.b	n.b	n.b.

AP-PE – Phenolic extract of frozen apple pomace, FD-AP-PE – Phenolic extract of freeze-dried apple pomace, AP-OE – Organic extract of frozen apple pomace, FD-AP-OE – Organic extract of freeze-dried apple pomace, B-PE-PVA – PVA biofilm with phenolic extract, B-PE-PEC – Pectin biofilm with phenolic extract, B-OE-PVA – PVA biofilm with organic extract, B-PE-PEC – Pectin biofilm with organic extract, n.b. – no bioactivity
